# Prenatal diagnosis of vasa previa and the course of the cord vessels contribute to the safety of cesarean sections: A case report

**DOI:** 10.1002/ccr3.2432

**Published:** 2019-09-27

**Authors:** Mihoko Aoki, Soichiro Obata, Mizuha Odagami, Etsuko Miyagi, Shigeru Aoki

**Affiliations:** ^1^ Perinatal Center for Maternity and Neonates Yokohama City University Medical Center Yokohama Japan; ^2^ Department of Obstetrics and Gynecology Yokohama City University Hospital Yokohama Japan

**Keywords:** cesarean section, prenatal diagnosis, ultrasonography, vasa previa

## Abstract

Vasa previa can occur even in cases without placental malposition and the precise diagnosis of vasa previa, and the course of the cord vessels contributes to a safe delivery. The color Doppler is a useful and easy‐to‐use device to confirm the presence of vasa previa.

## Graphical abstract

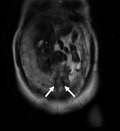

## 1 INTRODUCTION

Vasa previa is defined as a condition in which the cord vessels are present in the membranes covering the internal cervical os.^1^ The prevalence of vasa previa is approximately 1 in 2500 pregnancies, and its pregnancy outcomes are poor.^2^ Its prevalence is related to conditions of low‐lying placenta or abnormal placental morphology, such as bilobed or succenturiate lobe placentas in the lower uterine segment.^3^, ^4^, ^5^ Here, we report a case of vasa previa without placental malposition or abnormal placental morphology. Because the placenta was on the anterior wall of the uterus and the cord vessels were running on the anterior wall of the lower uterine segment, the fetus was safely delivered by cesarean section with the horizontal incision on the uterine fundus.

## 2 CASE

**Figure 1:**
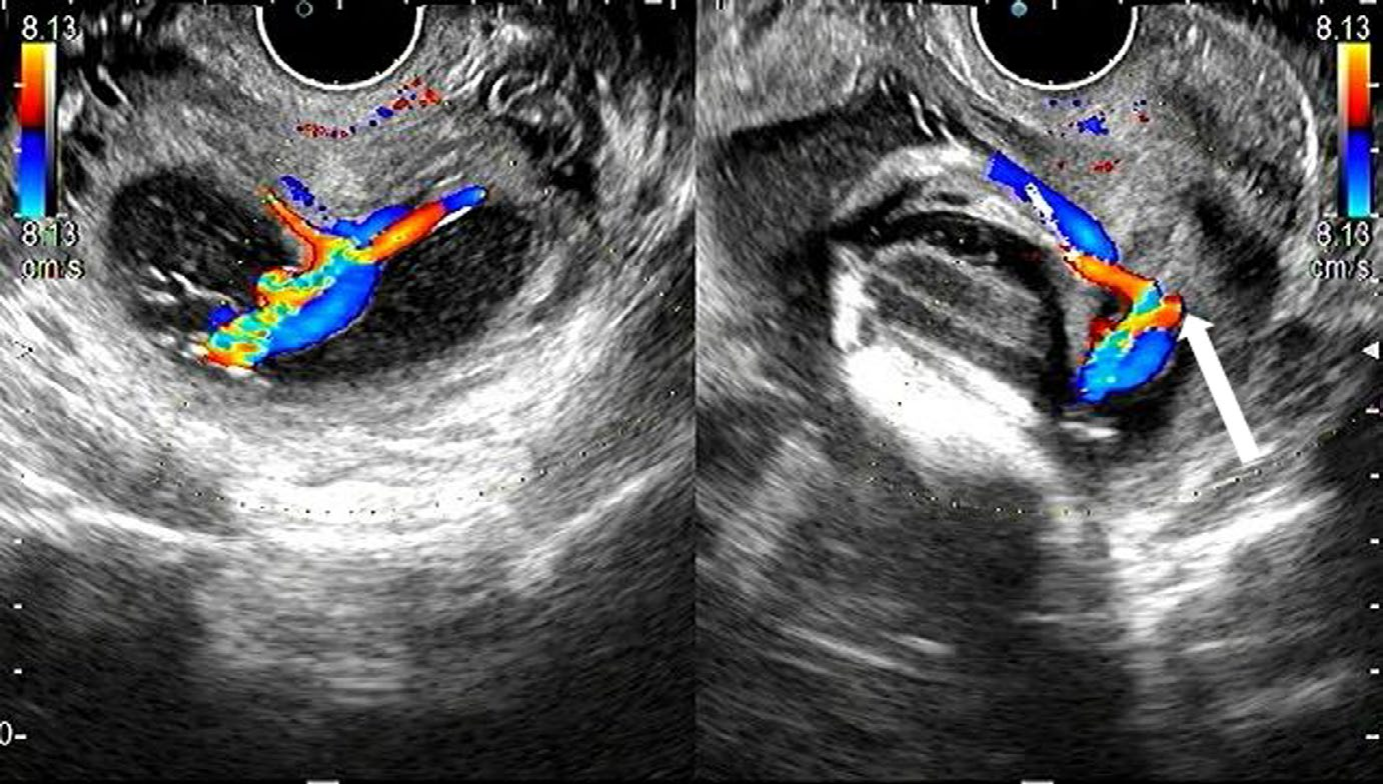
Transvaginal ultrasonography image taken at the first visit showing the vasa previa. The cord vessels are shown running on the anterior lower uterine segment and covering the internal cervical os (white arrow)

**Figure 2:**
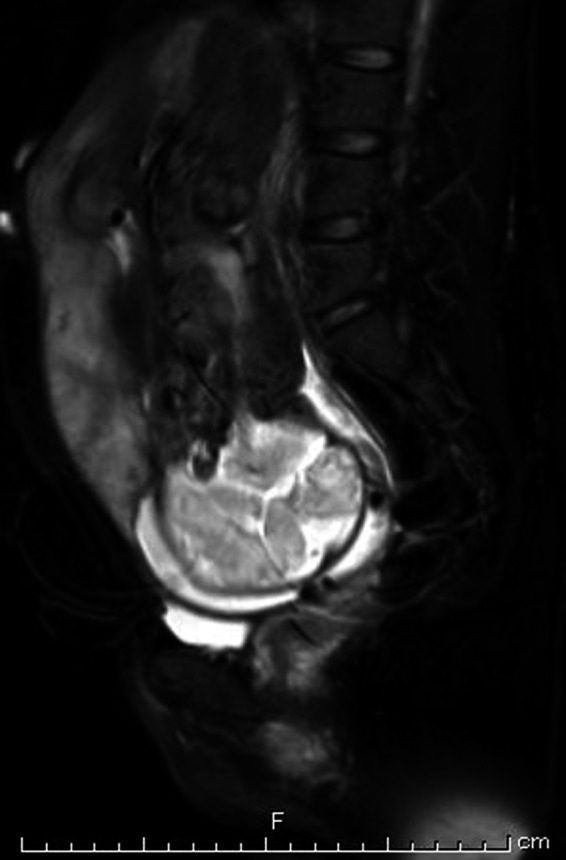
T2‐weighted MRI confirmed the normal placental position and the absence of abnormal placental morphology

**Figure 3:**
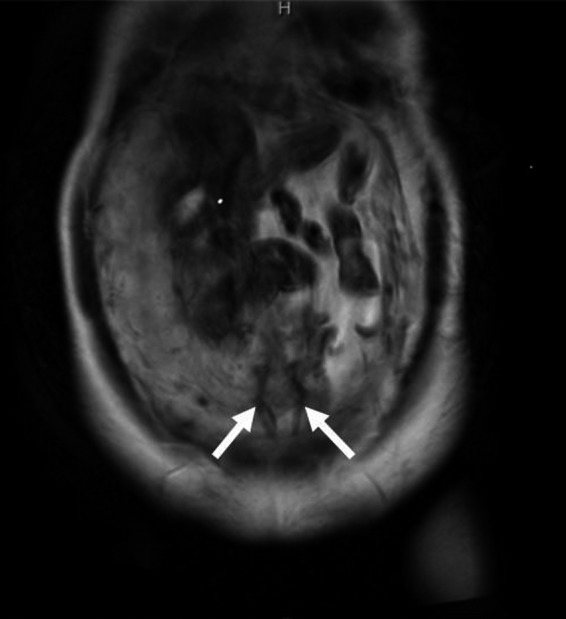
T2‐weighted MRI confirmed that the cord vessels were running on the anterior lower uterine segment. The white arrows indicate the cord vessels

**Figure 4:**
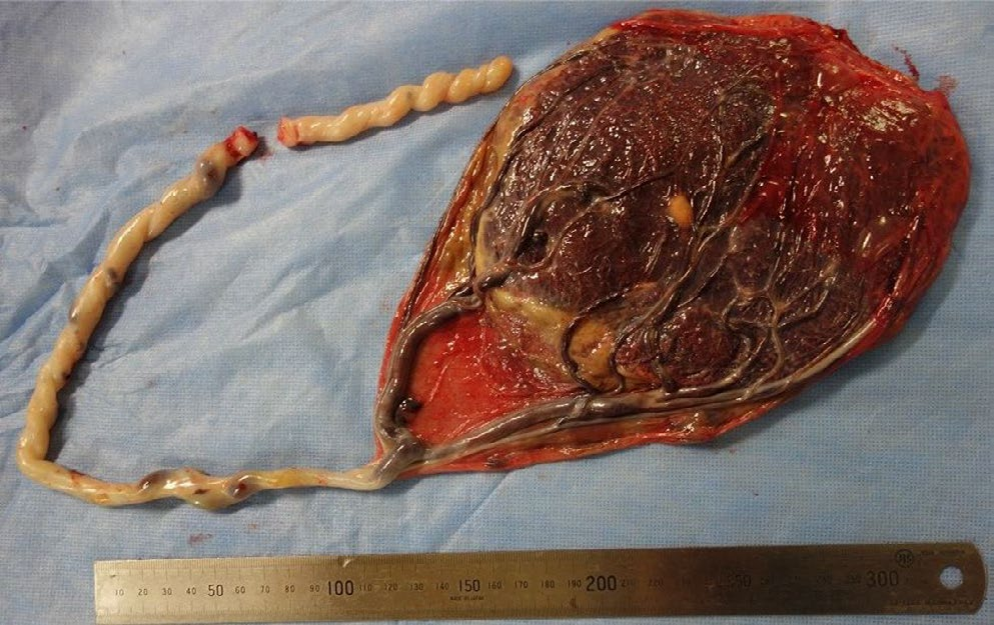
The placenta showed velamentous cord insertion and the length of the vessels running on the membranes was about 10 cm

Our case is a 24‐year‐old primigravida woman. She conceived spontaneously and her due date was confirmed by crown‐rump length measurements in the first trimester. At 18 weeks of gestation, there were no signs of a low‐lying placenta or placenta previa. At 24 weeks of gestation, she complained of vaginal bleeding and came to the clinic for a consultation. On examination, the patient was found to have slight intermittent bleeding. Transvaginal ultrasonography showed signs of vasa previa, and she was referred to our perinatal center. On ultrasonography, the placenta was seen to be on the anterior wall and there were no signs of placental malposition. The presence of vasa previa as well as velamentous umbilical cord insertion was confirmed by transvaginal ultrasonography, which demonstrated the cord vessels running on the anterior lower uterine segment and covering the internal cervical os (Figure 1). The vaginal bleeding ceased on examination, and it was found to be unrelated to the vasa previa. There was no fetal distress detected on fetal heart monitoring. She was hospitalized from 32 weeks and 5 days of gestation. At 33 weeks of gestation, the normal placental position as well as the absence of abnormal placental morphology was confirmed by an MRI scan (Figure 2). In addition, MRI confirmed the precise course of the cord vessels running on the anterior lower segment of the uterus (Figure 3). We performed a cesarean section at 35 weeks and 1 day of gestation. Because the cord vessels were running on the anterior lower uterine segment, the horizontal incision was made on the uterine fundus to avoid the rupture of the cord vessels. A male baby was delivered, and the neonate had a birth weight of 1836 g (small for gestational age) and Apgar scores of 8 and 9 at 1 and 5 minutes, respectively. The placenta showed velamentous cord insertion and the length of the vessels running on the membranes was about 10 cm (Figure 4). There were no problems in the postpartum period, and the patient was discharged with her baby 7 days after the cesarean section.

We explained the possibility of publishing this study as a case report to the patient, and her consent was obtained.

## 3 DISCUSSION

This case report describes a patient with vasa previa who did not have concomitant placental malposition. In a case of vasa previa with the placenta situated in the anterior normal position, the cord vessels may run in the anterior lower uterine segment. In such a case, the fetus can be safely delivered by a horizontal incision on the uterine fundus. The precise prenatal diagnosis of vasa previa and the course of the cord vessels contribute to a safe delivery.

In vasa previa, the cord vessels are present in the membranes covering the internal cervical os.^1^ The prevalence of vasa previa is approximately 1 in 2500 pregnancies^2^ and is related to either a low‐lying placenta or abnormal placental morphology, such as bilobed or succenturiate lobe placentas in the lower uterine segment.^3^, ^4^, ^5^ Velamentous umbilical cord insertion is also related to the occurrence of vasa previa.^2^ Therefore, in patients with velamentous umbilical cord insertion, low‐lying placenta, or abnormal placental morphology, the diagnosis of vasa previa must be considered.^6^, ^7^, ^8^ The American College of Obstetricians and Gynecologists also recommends the use of color Doppler in patients who are at a high risk for vasa previa.^9^ However, in some cases, it is difficult to determine the cord insertion and abnormal placental morphology.^10^, ^11^ In our patient, vasa previa was not accompanied by either a low‐lying placenta or abnormal placental morphology. Moreover, the velamentous cord insertion was not determined before the diagnosis of vasa previa. Therefore, even in cases without placental malposition or abnormal placental morphology, the possibility of vasa previa must be considered. It has been reported that cases of vasa previa with a prenatal diagnosis have a 91.6% neonatal survival rate, whereas those without a prenatal diagnosis have only a 43.6% neonatal survival rate.^12^ In addition, the transfusion rate in patients with a prenatal diagnosis was only 3.4% compared to 58.8% in those without a prenatal diagnosis.^13^ Therefore, prenatal diagnosis of vasa previa is very important.

In our case, because the cord vessels were running on the anterior lower uterine segment, we delivered the fetus with a horizontal incision on the uterine fundus to avoid the rupture of the cord vessels. In a case of vasa previa with placental attachment on the anterior uterine wall, the cord vessels may run on the anterior lower uterine segment. In such cases, there is a risk of rupture of cord vessels, if a horizontal incision is made in the lower uterine segment for the cesarean section. In our patient, the presence of vasa previa as well as the precise course of the cord vessels was confirmed by ultrasonography with color Doppler. The horizontal incision on the uterine fundus has a higher risk of uterine rupture or placenta accreta relative to that of the incision in the lower uterine segment,^14^ which instead has a higher risk of rupture of cord vessels and loss of massive fetal blood. Therefore, we chose to perform the horizontal incision on the uterine fundus. Confirming the precise course of the cord vessels helps in the safe delivery of the fetus.

Determining the presence of risk factors for vasa previa, such as velamentous cord insertion, a low‐lying placenta, or abnormal placental morphology, is difficult in some cases. In addition, there is no consensus for screening for vasa previa in low‐risk cases. However, measurement of the cervical length using transvaginal ultrasonography in midtrimester is usually performed. If we use the color Doppler concurrently when measuring the cervical length, it would be easy to confirm the presence of vasa previa and its diagnostic rate would increase. The color Doppler is a useful and easy‐to‐use device to confirm the presence of vasa previa. If the color Doppler is available, it should be used actively.

There is no consensus for the optimal timing of a cesarean section in patients with vasa previa. Previous reports have recommended performing a cesarean section at 34 to 37 weeks of gestation considering the immaturity of the fetus.^12^, ^15^ In our facility, we perform the cesarean section in patients with vasa previa at 35 weeks of gestation considering the maturity of the fetus and to avoid an emergent cesarean section. However, a cesarean section should be performed immediately if there is massive vaginal hemorrhage, an abnormality in the fetal heart monitoring, or rupture of membranes.

In conclusion, vasa previa results in poor pregnancy outcomes if not diagnosed prenatally, and therefore, even low‐risk cases must be screened for vasa previa. The course of the cord vessels must also be confirmed. Ultrasonography with color Doppler is a useful as well as convenient diagnostic method for the same. Prenatal confirmation of the presence of vasa previa as well as the course of the cord vessels contributes to the safe delivery of the baby.

## CONFLICT OF INTEREST

None declared.

## AUTHOR CONTRIBUTIONS

MA: Contributed to the finalization of the manuscript and performed the surgery. SO: Contributed to the first draft and finalization of the manuscript. MO: Performed the surgery. EM and SA: Supervised the case report.
